# Supplementary material for: Estimates and correlates of district-level maternal mortality ratio in India
Source: PLOS Glob Public Health. 2022 Jul 18;2(7):e0000441. doi: 10.1371/journal.pgph.0000441 (PMC10021851; doi:10.1371/journal.pgph.0000441)
Supplement: S1 Fig — (PDF) [file pgph.0000441.s001.pdf]

S 1 Fig Bivariate LISA (Cluster and Significance) maps depicting spatial clustering and spatial outliers of maternal mortality ratio by selected background characteristics in India

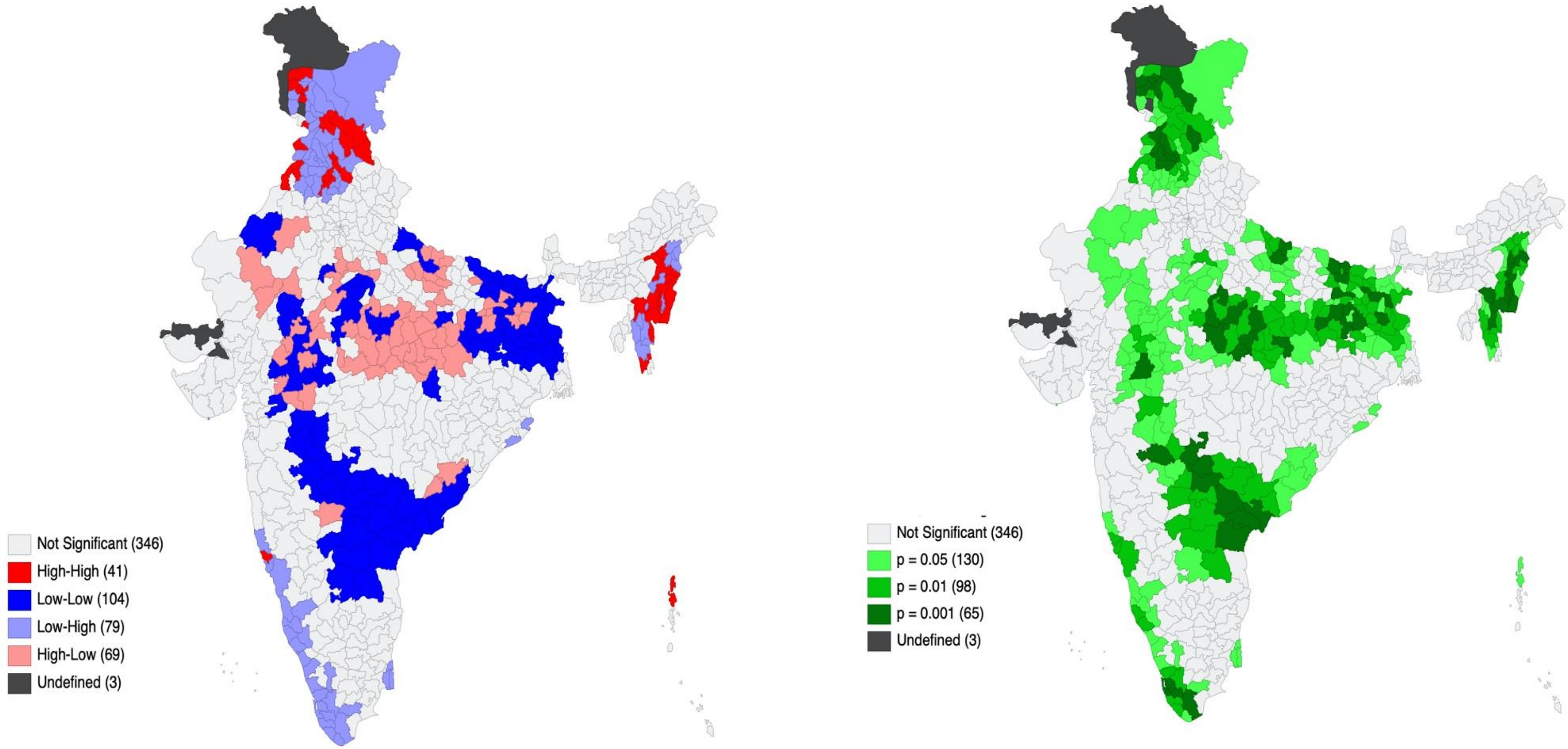

Bivariate LISA (Cluster and Significance) maps depicting spatial clustering and spatial outliers of maternal mortality ratio by mean age at marriage in India (Moran's I=0.000, p-value=0.498)

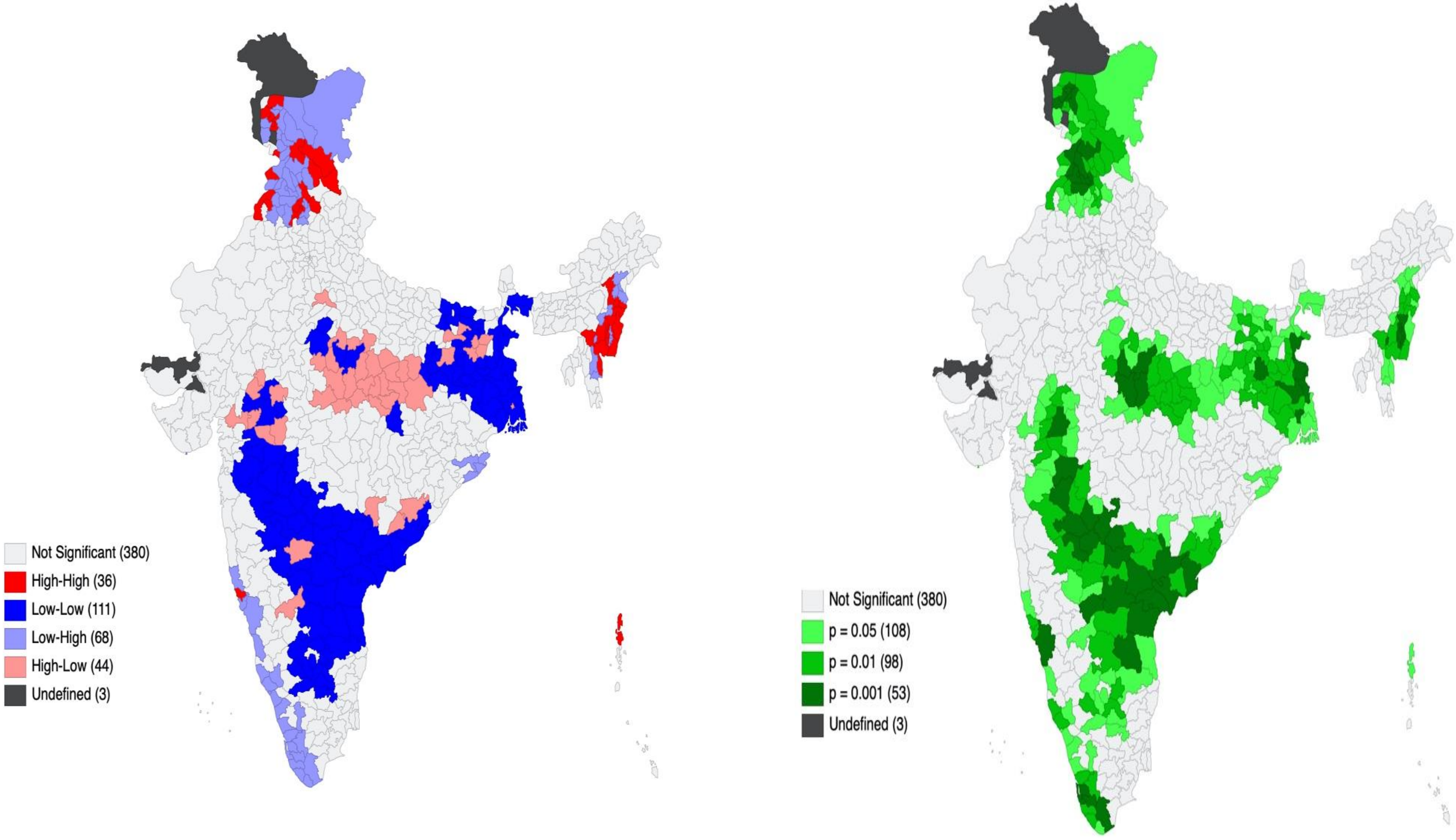

Bivariate LISA (Cluster and Significance) maps depicting spatial clustering and spatial outliers of maternal mortality ratio by mean age at first birth in India (Moran's I=0.035, p-value=0.0350)

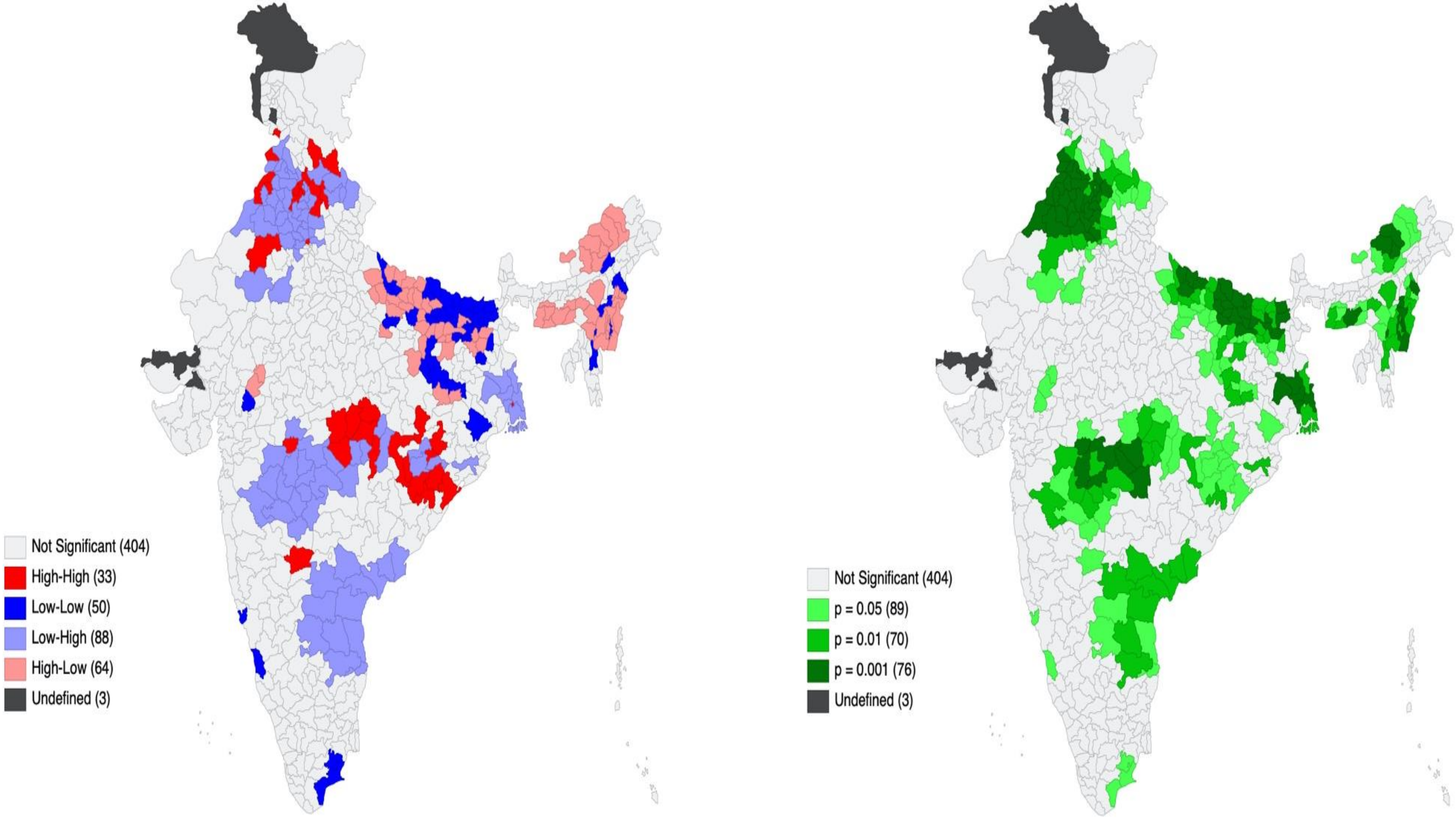

Bivariate LISA (Cluster and Significance) maps depicting spatial clustering and spatial outliers of maternal mortality ratio by contraception use in India (Moran's I=-0.166, p-value=0.001)

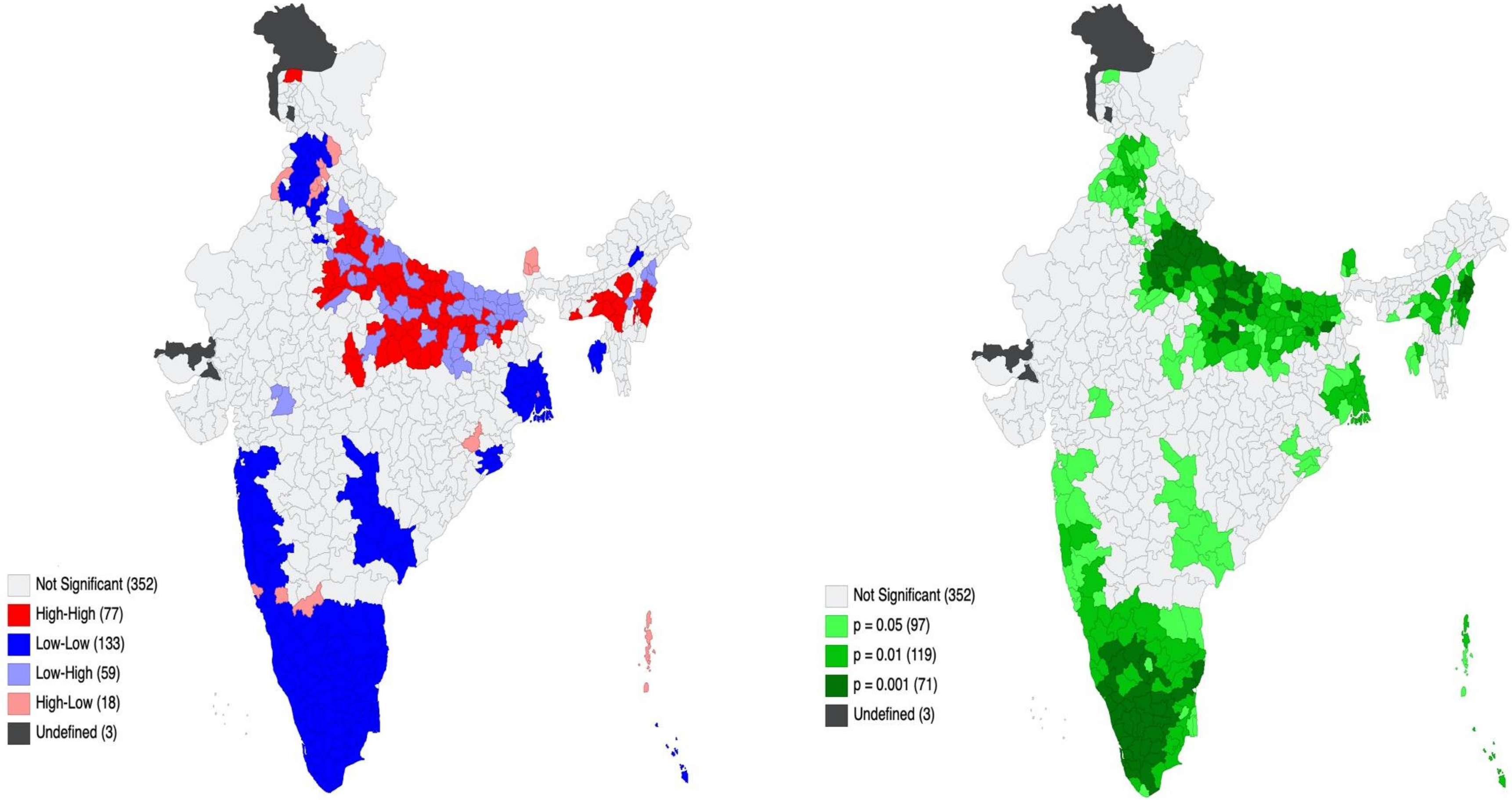

Bivariate LISA (Cluster and Significance) maps depicting spatial clustering and spatial outliers of maternal mortality ratio by mean number of children ever born in India (Moran's I=0.258, p-value=0.001)

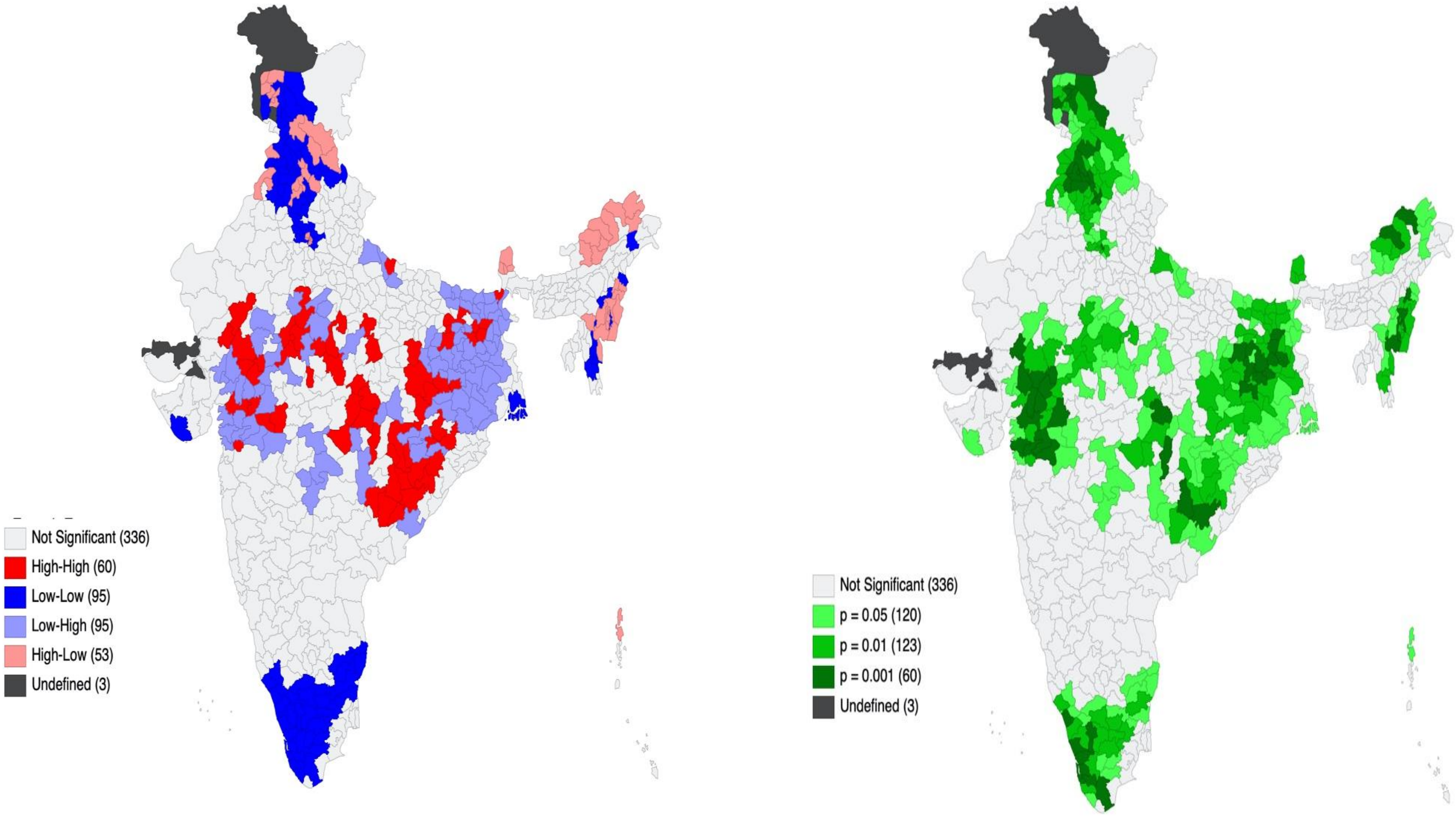

Bivariate LISA (Cluster and Significance) maps depicting spatial clustering and spatial outliers of maternal mortality ratio by percent of women underweight in India (Moran's I=0.024, p-value=0.091)

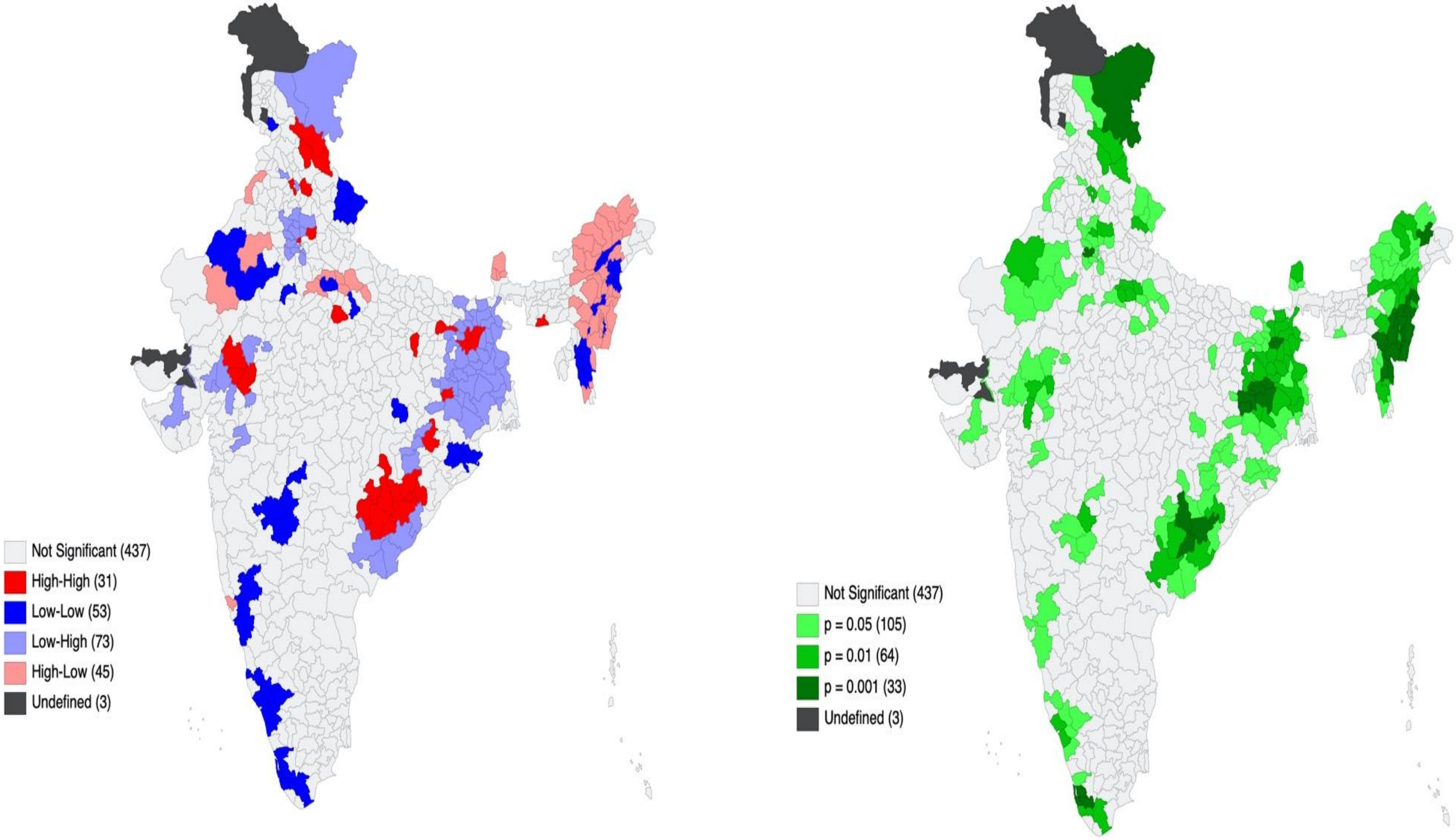

Bivariate LISA (Cluster and Significance) maps depicting spatial clustering and spatial outliers of maternal mortality ratio by percent of anaemic women mean in India (Moran's I=-0.045, p-value=0.019)

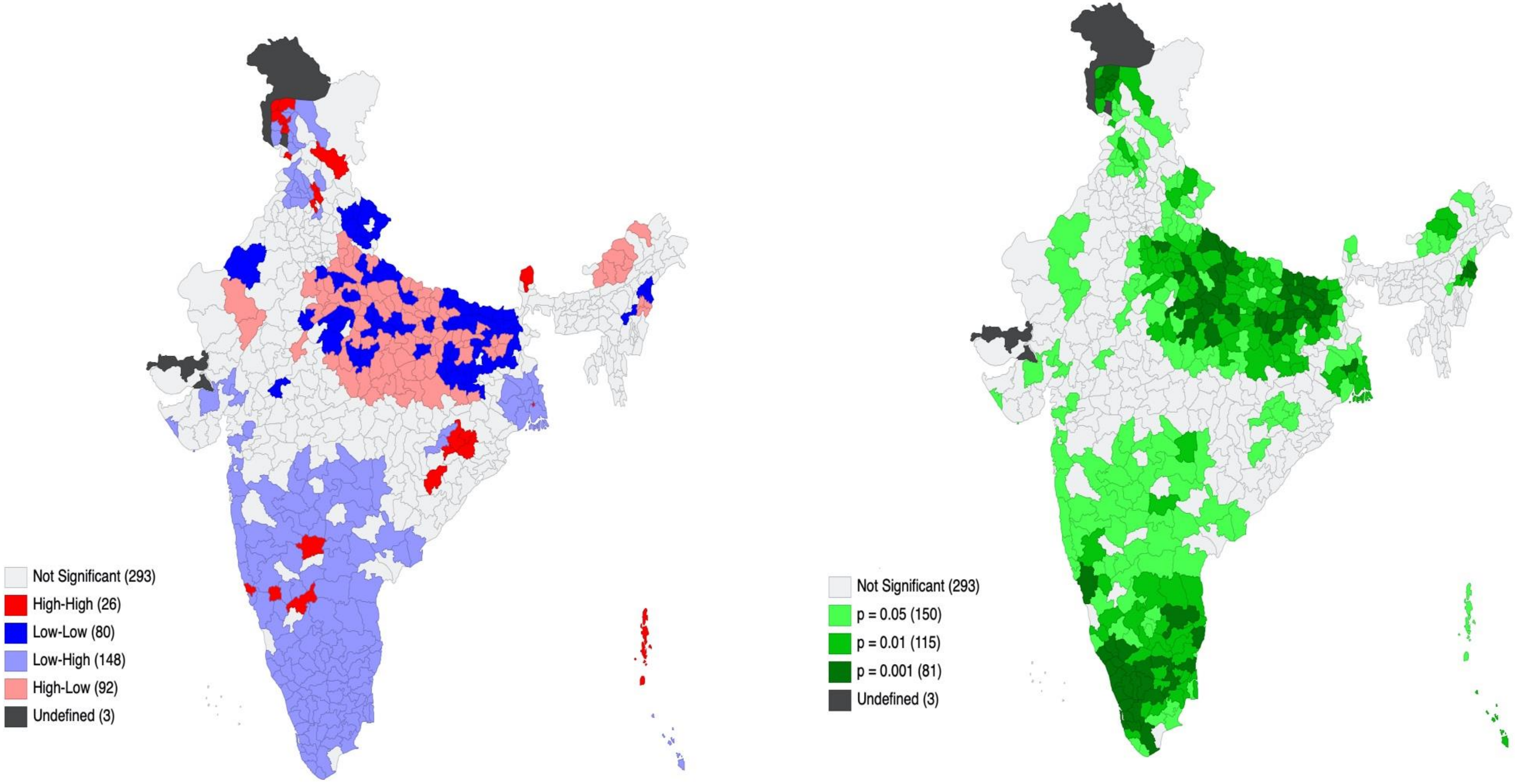

Bivariate LISA (Cluster and Significance) maps depicting spatial clustering and spatial outliers of maternal mortality ratio by percent of women receiving four or more Antenatal care in India (Moran's I=-0.241, p-value=0.001)

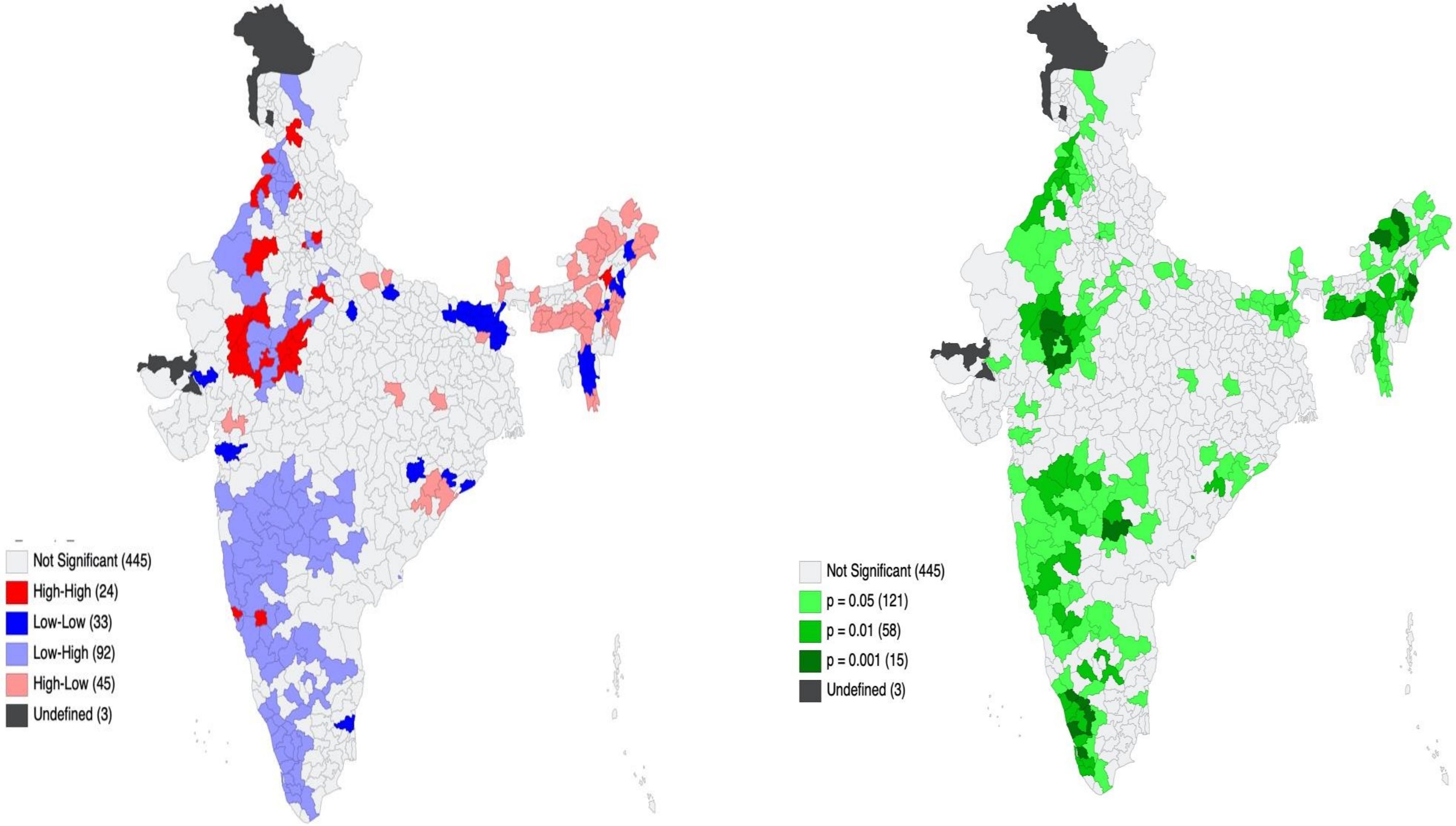

Bivariate LISA (Cluster and Significance) maps depicting spatial clustering and spatial outliers of maternal mortality ratio by percent women receiving Postnatal care in India (Moran's I=-0.168, p-value=0.001)

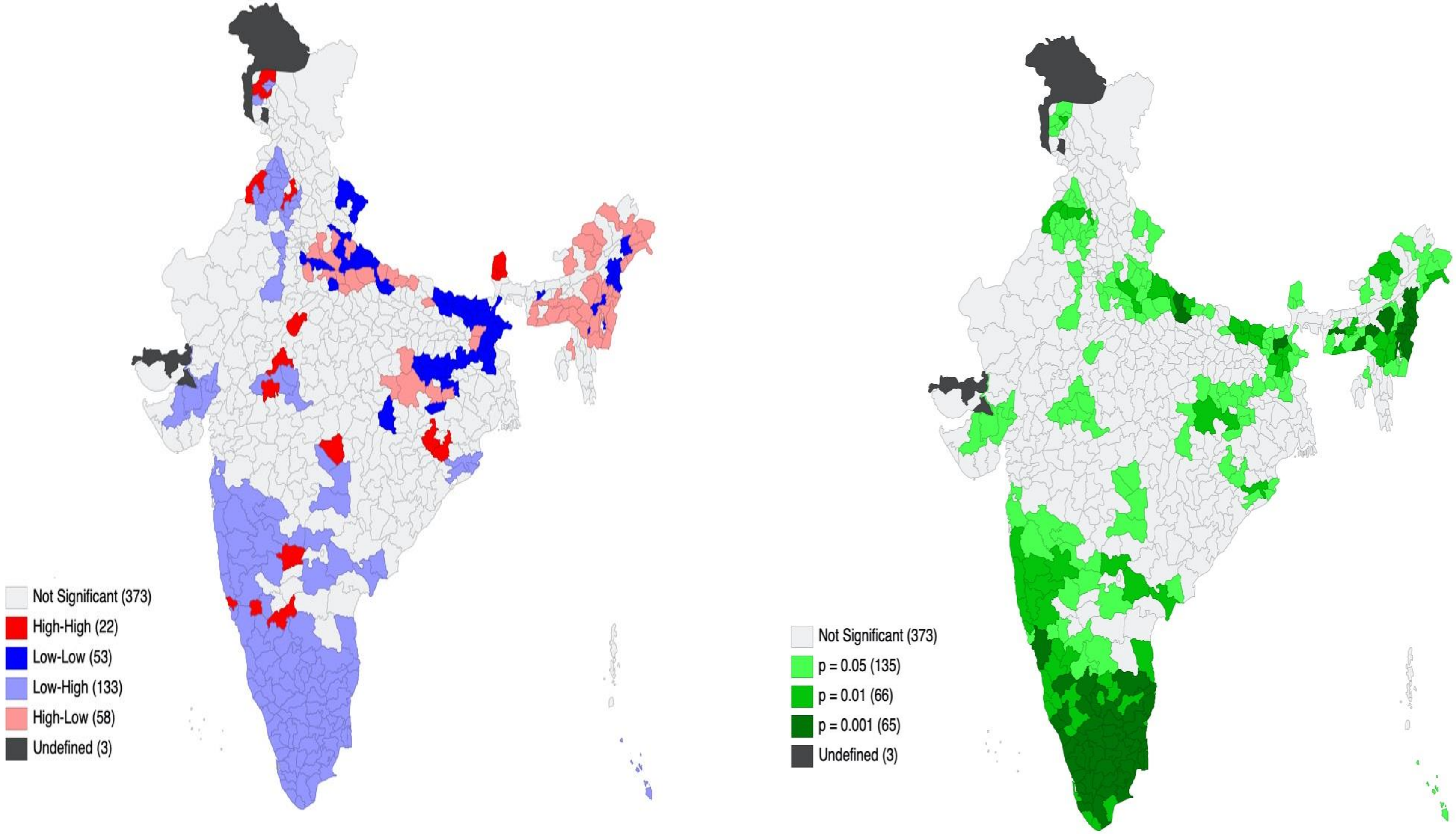

Bivariate LISA (Cluster and Significance) maps depicting spatial clustering and spatial outliers of maternal mortality ratio by percent of institutional delivery in India (Moran's I=-0.233, p-value=0.001)

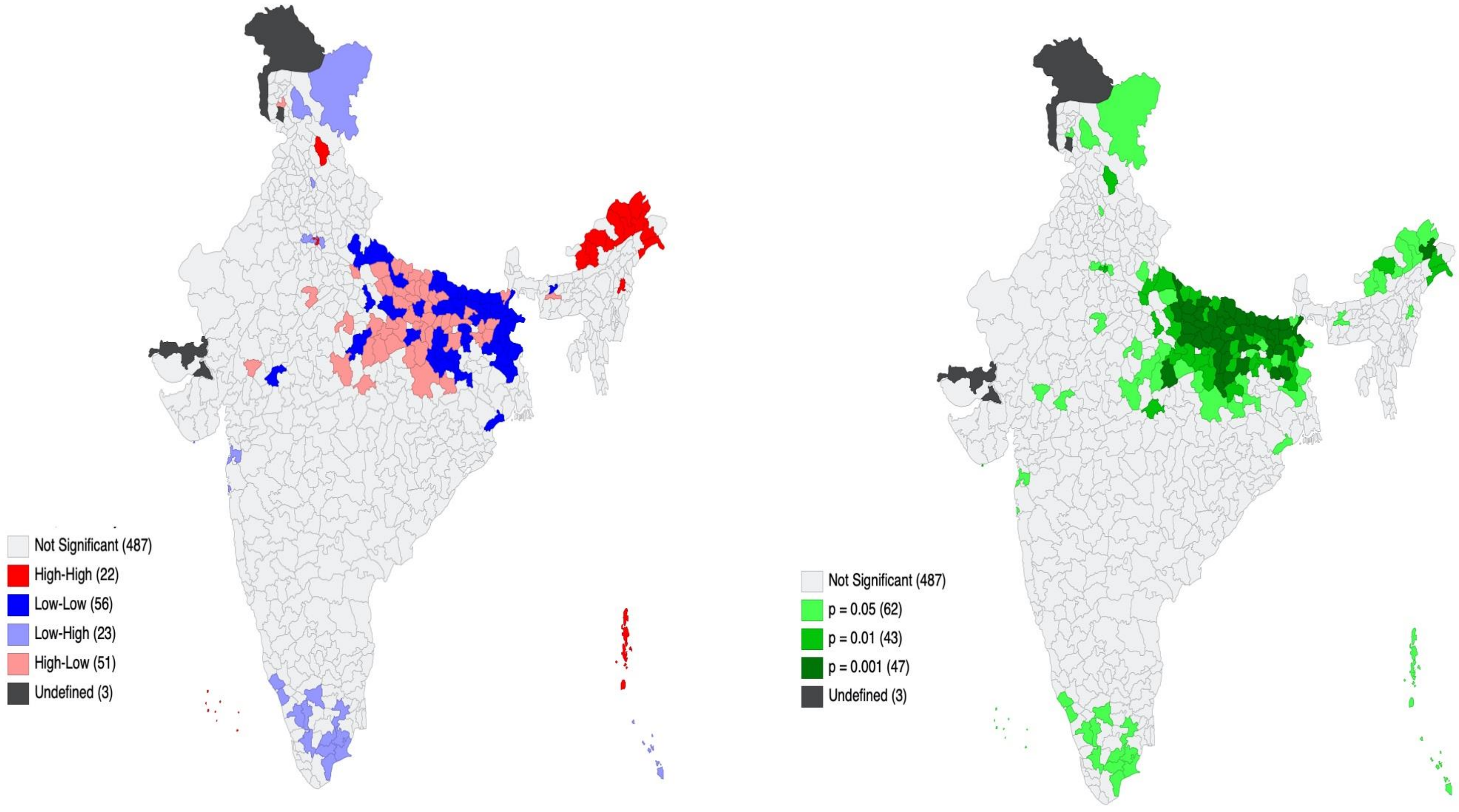

Bivariate LISA (Cluster and Significance) maps depicting spatial clustering and spatial outliers of maternal mortality ratio by healthcare index in India (Moran's I=-0.042, p-value=0.001)

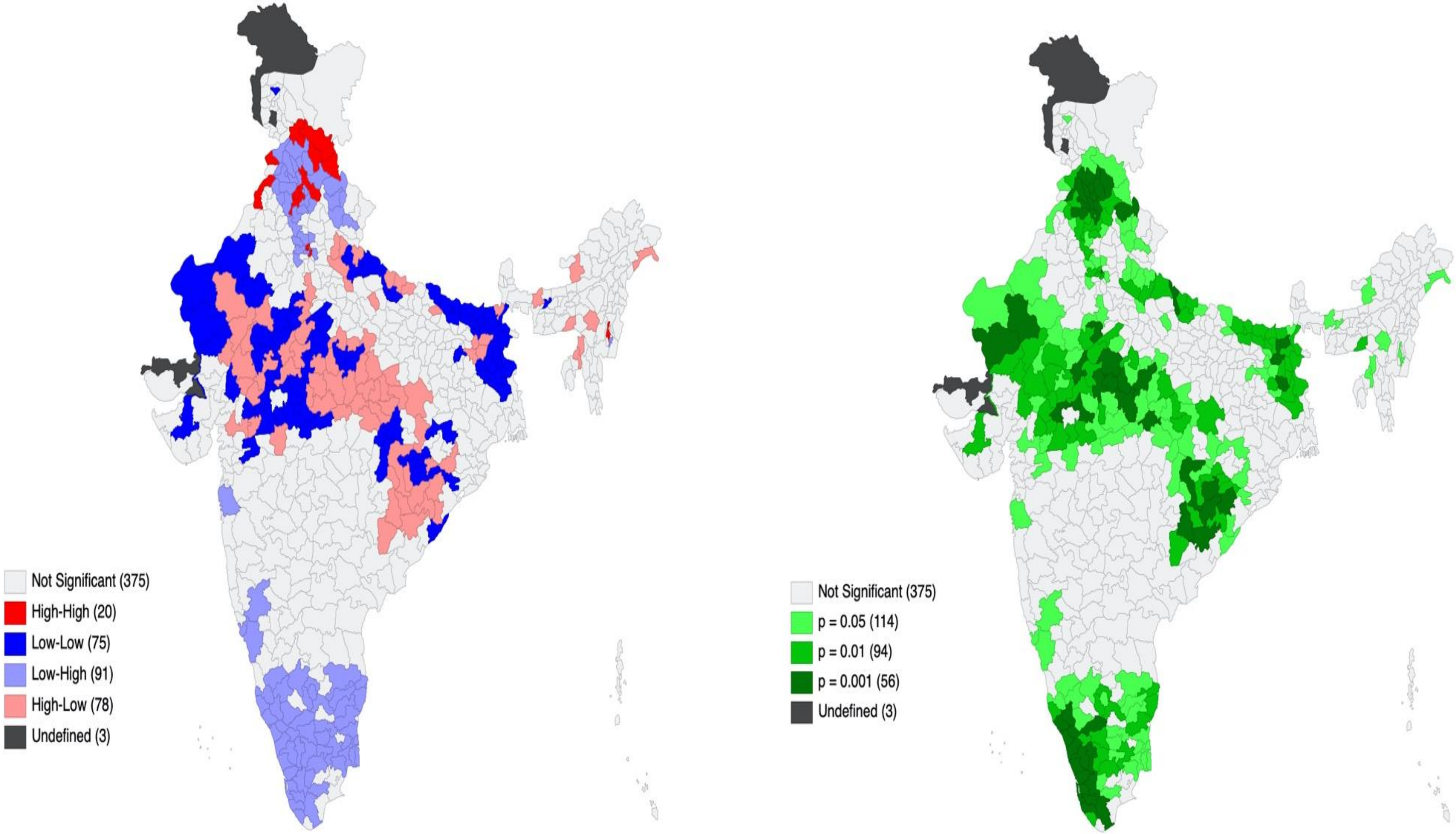

Bivariate LISA (Cluster and Significance) maps depicting spatial clustering and spatial outliers of maternal mortality ratio by percent women with ten or more years of schooling in India (Moran's I=-0.017, p-value=0.001)

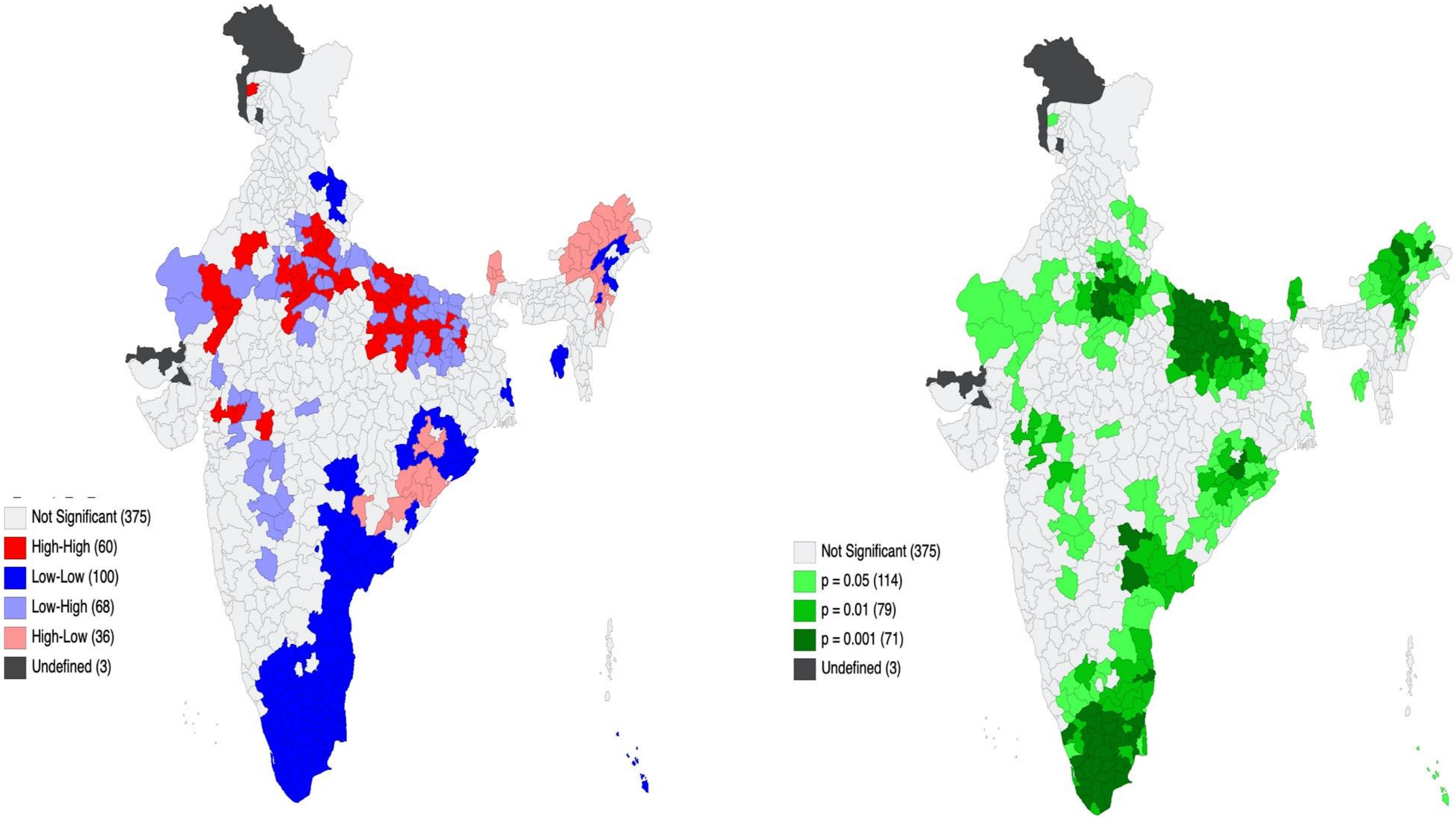

Bivariate LISA (Cluster and Significance) maps depicting spatial clustering and spatial outliers of maternal mortality ratio by average household size in India (Moran's I=0.110, p-value=0.001)

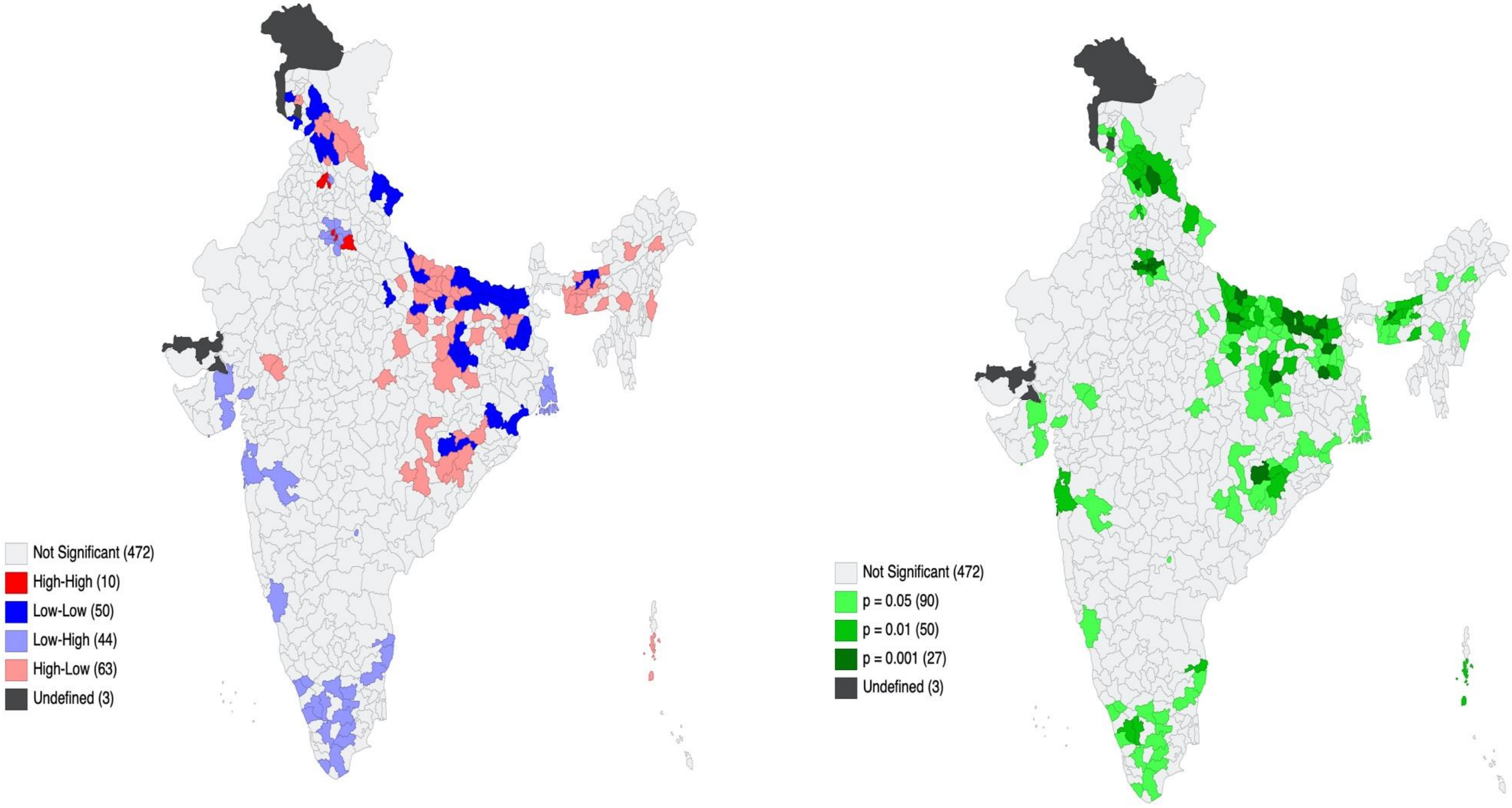

Bivariate LISA (Cluster and Significance) maps depicting spatial clustering and spatial outliers of maternal mortality ratio by percent women residing in urban areas in India (Moran's I=-0.152, p-value=0.001)

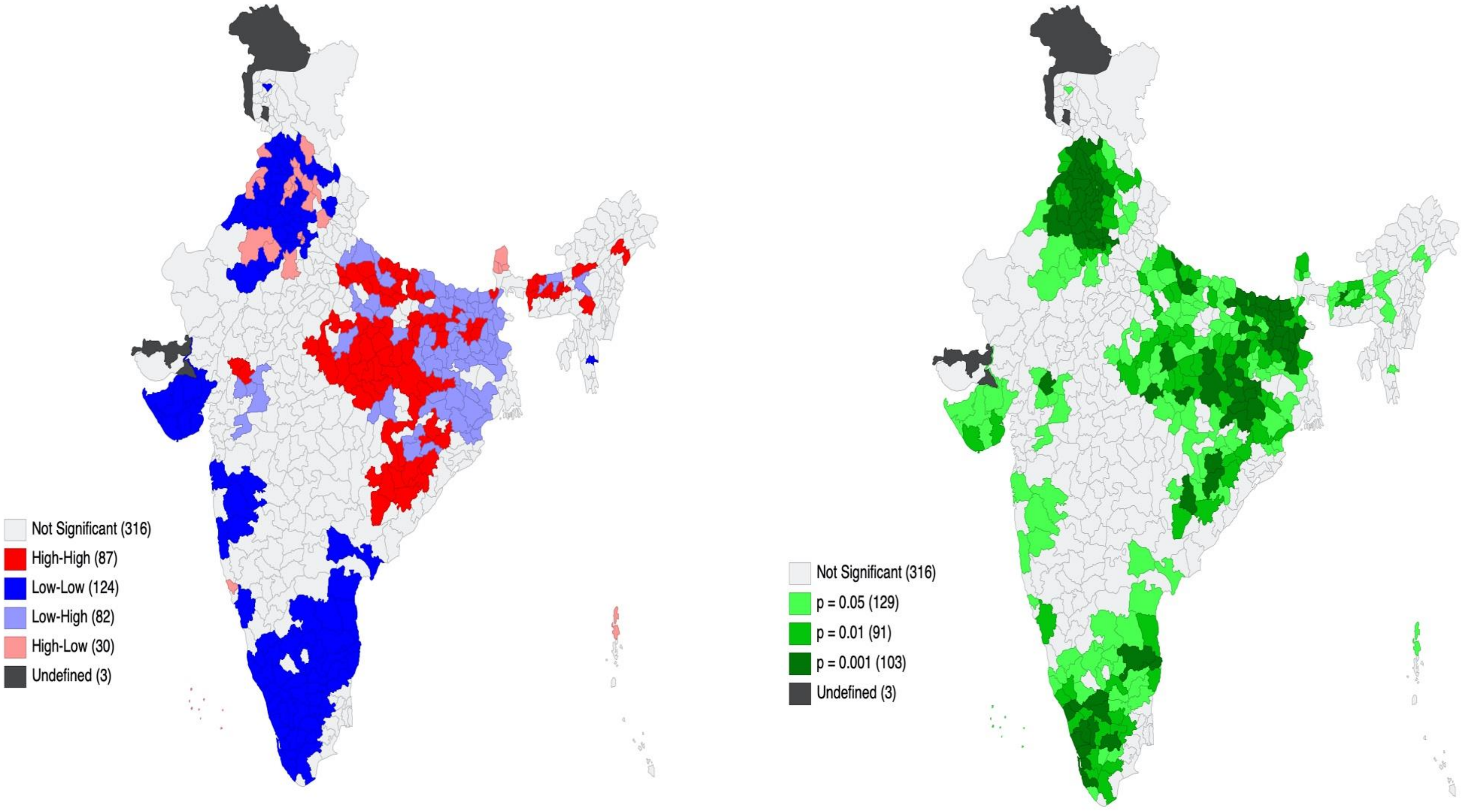

Bivariate LISA (Cluster and Significance) maps depicting spatial clustering and spatial outliers of maternal mortality ratio by percent poor in India (Moran's I=0.215, p-value=0.001)

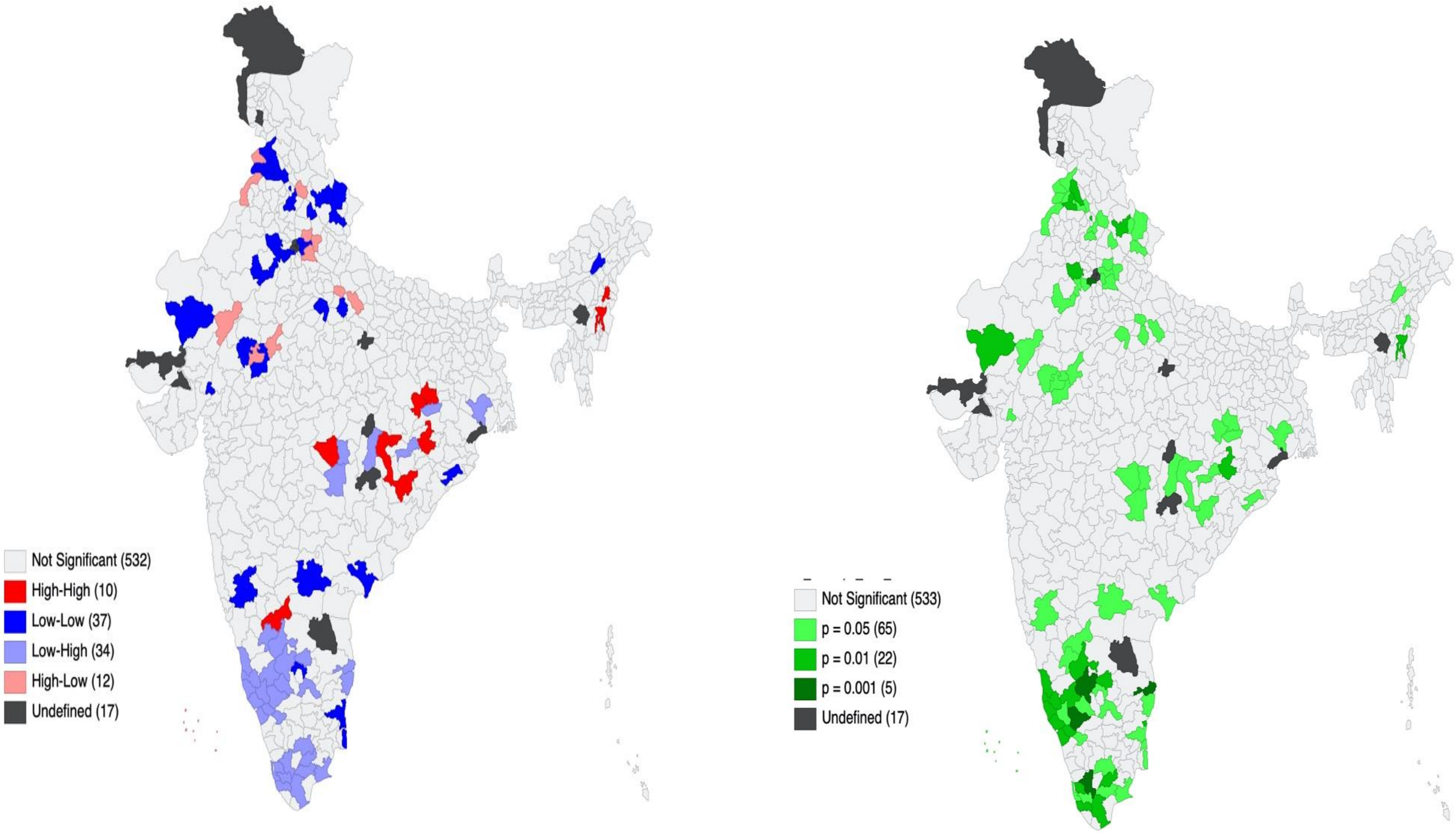

Bivariate LISA (Cluster and Significance) maps depicting spatial clustering and spatial outliers of maternal mortality ratio by sex ratio at birth in India (Moran's I=-0.017, p-value=0.189)
