# Supplementary material for: Estimates and correlates of district-level maternal mortality ratio in India
Source: PLOS Glob Public Health. 2022 Jul 18;2(7):e0000441. doi: 10.1371/journal.pgph.0000441 (PMC10021851; doi:10.1371/journal.pgph.0000441)
Supplement: S1 Table — (PDF) [file pgph.0000441.s002.pdf]

S1 Table. Strengthening the reporting of observational studies or cross-sectional studies in epidemiology (STROBE Checklist)

	Item No	Recommendation	Page No
**Title and abstract**	1	(*a*) Indicate the study's design with a commonly used term in the title or the abstract	Page no. 1
		(*b*) Provide in the abstract an informative and balanced summary of what was done and what was found	Page no. 1
**Introduction**			
Background/rationale	2	Explain the scientific background and rationale for the investigation being reported	Page no. 3
Objectives	3	State-specific objectives, including any prespecified hypotheses	Page no. 4
**Methods**			
Study design	4	Present key elements of study design early in the paper	Page no. 4
Setting	5	Describe the setting, locations, and relevant dates, including periods of recruitment, exposure, follow-up, and data collection	Page no. 4
Participants	6	(*a*) Give the eligibility criteria, and the sources and methods of selection of participants	Page no. 4
Variables	7	Clearly define all outcomes, exposures, predictors, potential confounders, and effect modifiers. Give diagnostic criteria, if applicable	Page no. 5
Data sources/ measurement	8*	For each variable of interest, give sources of data and details of methods of assessment (measurement). Describe comparability of assessment methods if there is more than one group	Page no. 4 and 5
Bias	9	Describe any efforts to address potential sources of bias	Page no. 4
Study size	10	Explain how the study size was arrived at	Page no. 4
Quantitative variables	11	Explain how quantitative variables were handled in the analyses. If applicable, describe which groupings were chosen and why	Page no. 5
Statistical methods	12	(*a*) Describe all statistical methods, including those used to control for confounding	Page no. 5 and 6
		(*b*) Describe any methods used to examine subgroups and interactions	Page no. 5 and 6
		(*c*) Explain how missing data were addressed	Page no. 5 and 6
		(*d*) If applicable, describe analytical methods taking account of sampling strategy	Page no. 5 and 6
		(*e*) Describe any sensitivity analyses	Page no. 8
**Results**			
Participants	13*	(a) Report numbers of individuals at each stage of study—eg numbers potentially eligible, examined for eligibility, confirmed eligible, included in the study, completing follow-up, and analysed	NA
		(b) Give reasons for non-participation at each stage	NA
		(c) Consider use of a flow diagram	NA

Descriptive data	14*	(a) Give characteristics of study participants (eg demographic, clinical, social) and information on exposures and potential confounders	Page no. 6, Table 1 and Table 2
		(b) Indicate number of participants with missing data for each variable of interest	Page no. 6, Table 1.
Outcome data	15*	Report numbers of outcome events or summary measures	Page no. 6, Table 1.
Main results	16	(*a*) Give unadjusted estimates and, if applicable, confounder-adjusted estimates and their precision (eg, 95% confidence interval). Make clear which confounders were adjusted for and why they were included	Page no. 6 to Page no. 9. Table 3 and Figure 1, 2, 3
		(*b*) Report category boundaries when continuous variables were categorized	NA
		(*c*) If relevant, consider translating estimates of relative risk into absolute risk for a meaningful time period	NA
Other analyses	17	Report other analyses done—eg analyses of subgroups and interactions, and sensitivity analyses	Page no. 8, Figure 4 and Figure 5.
**Discussion**			
Key results	18	Summarise key results with reference to study objectives	Page no. 10
Limitations	19	Discuss limitations of the study, taking into account sources of potential bias or imprecision. Discuss both direction and magnitude of any potential bias	Page no. 10
Interpretation	20	Give a cautious overall interpretation of results considering objectives, limitations, multiplicity of analyses, results from similar studies, and other relevant evidence	Page no. 10
Generalisability	21	Discuss the generalisability (external validity) of the study results	Page no. 10 and 11
**Other information**			
Funding	22	Give the source of funding and the role of the funders for the present study and, if applicable, for the original study on which the present article is based	Page no. 11.

*Give information separately for exposed and unexposed groups.

Note: An Explanation and Elaboration article discusses each checklist item and gives methodological background and published examples of transparent reporting. The STROBE checklist is best used in conjunction with this article (freely available on the Web sites of PLoS Medicine at http://www.plosmedicine.org/, Annals of Internal Medicine at http://www.annals.org/, and Epidemiology at http://www.epidem.com/). Information on the STROBE Initiative is available at www.strobe-statement.org.
