# Supplementary material for: Estimates and correlates of district-level maternal mortality ratio in India
Source: PLOS Glob Public Health. 2022 Jul 18;2(7):e0000441. doi: 10.1371/journal.pgph.0000441 (PMC10021851; doi:10.1371/journal.pgph.0000441)
Supplement: S2 Table — (PDF) [file pgph.0000441.s003.pdf]

S2 Table. State-wise estimates of MMR from SRS and HMIS

Sr. No	States	SRS (2014-16)	SRS (2016-18)	HMIS (2017-2019)
1	A & N Islands			275
2	Andhra Pradesh	74	65	64
3	Arunachal Pradesh			284
4	Assam	237	215	209
5	Bihar	165	149	164
6	Chandigarh			15
7	Chhattisgarh*		159	144
8	Dadra & Nagar Haveli			61
9	Daman & Diu			48
10	Delhi			162
11	Goa			91
12	Gujarat	91	75	76
13	Haryana	101	91	90
14	Himachal Pradesh			127
15	Jammu & Kashmir			151
16	Jharkhand*		71	78
17	Karnataka	108	92	85
18	Kerala	46	43	44
19	Lakshadweep			208
20	Madhya Pradesh	173	173	179
21	Maharashtra	61	46	40
22	Manipur			282
23	Meghalaya			266
24	Mizoram			131
25	Nagaland			143
26	Odisha	180	150	138
27	Puducherry			41
28	Punjab	122	129	143
29	Rajasthan	199	164	162
30	Sikkim			228
31	Tamil Nadu	66	60	56
32	Telangana	81	63	53
33	Tripura			119
34	Uttar Pradesh	201	197	208
35	Uttarakhand*		99	107
36	West Bengal	101	98	100
	INDIA	130	113	122

*Separate MMR estimates for these states are not available as they were merged with their parent states for SRS bulletin 2014-16
