# Supplementary material for: Estimates and correlates of district-level maternal mortality ratio in India
Source: PLOS Glob Public Health. 2022 Jul 18;2(7):e0000441. doi: 10.1371/journal.pgph.0000441 (PMC10021851; doi:10.1371/journal.pgph.0000441)
Supplement: S3 Table — (PDF) [file pgph.0000441.s004.pdf]

S3 Table. District-wise Estimates of MMR from HMIS

Sr. No.	District	State/Union Territory	MMR
1	Nicobars	Andaman & Nicobar	101
2	North & Middle Andaman	Andaman & Nicobar	605
3	South Andaman	Andaman & Nicobar	159
4	Anantapur	Andhra Pradesh	45
5	Chittoor	Andhra Pradesh	57
6	East Godavari	Andhra Pradesh	79
7	Guntur	Andhra Pradesh	89
8	Krishna	Andhra Pradesh	74
9	Kurnool	Andhra Pradesh	87
10	Prakasam	Andhra Pradesh	38
11	Sri Potti Sriramulu Nellore	Andhra Pradesh	40
12	Srikakulam	Andhra Pradesh	32
13	Visakhapatnam	Andhra Pradesh	129
14	Vizianagaram	Andhra Pradesh	38
15	West Godavari	Andhra Pradesh	29
16	Y.S.R.	Andhra Pradesh	40
17	Anjaw	Arunachal Pradesh	0
18	Dibang Valley	Arunachal Pradesh	0
19	Kurung Kumey	Arunachal Pradesh	0
20	Lohit	Arunachal Pradesh	184
21	Lower Dibang Valley	Arunachal Pradesh	336
22	Changlang	Arunachal Pradesh	0
23	East Kameng	Arunachal Pradesh	284
24	East Siang	Arunachal Pradesh	403
25	Lower Subansiri	Arunachal Pradesh	0
26	Papum Pare	Arunachal Pradesh	298
27	Tawang	Arunachal Pradesh	364
28	Tirap	Arunachal Pradesh	1671
29	Upper Siang	Arunachal Pradesh	0
30	Upper Subansiri	Arunachal Pradesh	247
31	West Kameng	Arunachal Pradesh	256
32	West Siang	Arunachal Pradesh	0
33	Baksa	Assam	107
34	Barpeta	Assam	150
35	Bongaigaon	Assam	139
36	Cachar	Assam	431
37	Chirang	Assam	170
38	Darrang	Assam	120
39	Dhemaji	Assam	109
40	Dhubri	Assam	186
41	Dibrugarh	Assam	379
42	Dima Hasao	Assam	232
43	Goalpara	Assam	179
44	Golaghat	Assam	296
45	Hailakandi	Assam	169
46	Jorhat	Assam	170
47	Kamrup	Assam	409

48	Kamrup Metropolitan	Assam	143
49	Karbi Anglong	Assam	200
50	Karimganj	Assam	310
51	Kokrajhar	Assam	365
52	Lakhimpur	Assam	73
53	Morigaon	Assam	142
54	Nagaon	Assam	134
55	Nalbari	Assam	88
56	Sivasagar	Assam	141
57	Sonitpur	Assam	246
58	Tinsukia	Assam	81
59	Udalguri	Assam	225
60	Araria	Bihar	103
61	Arwal	Bihar	452
62	Aurangabad	Bihar	98
63	Banka	Bihar	425
64	Begusarai	Bihar	61
65	Bhagalpur	Bihar	787
66	Bhojpur	Bihar	175
67	Buxar	Bihar	312
68	Darbhanga	Bihar	39
69	Gaya	Bihar	163
70	Gopalganj	Bihar	145
71	Jamui	Bihar	197
72	Jehanabad	Bihar	245
73	Kaimur (Bhabua)	Bihar	221
74	Katihar	Bihar	64
75	Khagaria	Bihar	26
76	Kishanganj	Bihar	190
77	Lakhisarai	Bihar	87
78	Madhepura	Bihar	25
79	Madhubani	Bihar	112
80	Munger	Bihar	241
81	Muzaffarpur	Bihar	83
82	Nalanda	Bihar	69
83	Nawada	Bihar	50
84	Pashchim Champaran	Bihar	64
85	Patna	Bihar	544
86	Purba Champaran	Bihar	136
87	Purnia	Bihar	122
88	Rohtas	Bihar	137
89	Saharsa	Bihar	44
90	Samastipur	Bihar	181
91	Saran	Bihar	59
92	Sheikhpura	Bihar	103
93	Sheohar	Bihar	121
94	Sitamarhi	Bihar	42
95	Siwan	Bihar	119
96	Supaul	Bihar	140
97	Vaishali	Bihar	47

98	Chandigarh	Chandigarh	15
99	Bastar	Chhattisgarh	248
100	Bijapur	Chhattisgarh	423
101	Bilaspur	Chhattisgarh	121
102	Dakshin Bastar Dantewada	Chhattisgarh	247
103	Dhamtari	Chhattisgarh	275
104	Durg	Chhattisgarh	98
105	Janjgir - Champa	Chhattisgarh	34
106	Jashpur	Chhattisgarh	176
107	Kabeerdham	Chhattisgarh	89
108	Korba	Chhattisgarh	121
109	Koriya	Chhattisgarh	234
110	Mahasamund	Chhattisgarh	136
111	Narayanpur	Chhattisgarh	184
112	Raigarh	Chhattisgarh	202
113	Raipur	Chhattisgarh	143
114	Rajnandgaon	Chhattisgarh	164
115	Surguja	Chhattisgarh	211
116	Uttar Bastar Kanker	Chhattisgarh	220
117	Dadra and Nagar Haveli	Dadra and Nagar Haveli	61
118	Daman	Daman & Diu	22
119	Diu	Daman & Diu	122
120	Central	Delhi	151
121	East	Delhi	270
122	New	Delhi	347
123	North	Delhi	93
124	North East	Delhi	153
125	North West	Delhi	198
126	South	Delhi	163
127	South West	Delhi	32
128	West	Delhi	181
129	North Goa	Goa	152
130	South Goa	Goa	15
131	Ahmadabad	Gujarat	70
132	Amreli	Gujarat	46
133	Anand	Gujarat	112
134	Banas Kantha	Gujarat	46
135	Bharuch	Gujarat	83
136	Bhavnagar	Gujarat	55
137	Dohad	Gujarat	50
138	Gandhinagar	Gujarat	51
139	Jamnagar	Gujarat	106
140	Junagadh	Gujarat	31
141	Kachchh	Gujarat	95
142	Kheda	Gujarat	49
143	Mahesana	Gujarat	62
144	Narmada	Gujarat	80
145	Navsari	Gujarat	58
146	Panch Mahals	Gujarat	101
147	Patan	Gujarat	80

148	Porbandar	Gujarat	78
149	Rajkot	Gujarat	69
150	Sabar Kantha	Gujarat	60
151	Surat	Gujarat	54
152	Surendranagar	Gujarat	71
153	Tapi	Gujarat	84
154	The Dangs	Gujarat	156
155	Vadodara	Gujarat	195
156	Valsad	Gujarat	62
157	Ambala	Haryana	58
158	Bhiwani	Haryana	105
159	Faridabad	Haryana	55
160	Fatehabad	Haryana	121
161	Gurgaon	Haryana	105
162	Hisar	Haryana	86
163	Jhajjar	Haryana	98
164	Jind	Haryana	93
165	Kaithal	Haryana	74
166	Karnal	Haryana	125
167	Kurukshetra	Haryana	82
168	Mahendragarh	Haryana	87
169	Mewat	Haryana	111
170	Palwal	Haryana	120
171	Panchkula	Haryana	35
172	Panipat	Haryana	101
173	Rewari	Haryana	59
174	Rohtak	Haryana	117
175	Sirsa	Haryana	88
176	Sonipat	Haryana	67
177	Yamunanagar	Haryana	93
178	Bilaspur	Himachal Pradesh	183
179	Chamba	Himachal Pradesh	150
180	Hamirpur	Himachal Pradesh	64
181	Kangra	Himachal Pradesh	103
182	Kinnaur	Himachal Pradesh	0
183	Kullu	Himachal Pradesh	223
184	Lahul & Spiti	Himachal Pradesh	0
185	Mandi	Himachal Pradesh	92
186	Shimla	Himachal Pradesh	109
187	Sirmaur	Himachal Pradesh	219
188	Solan	Himachal Pradesh	167
189	Una	Himachal Pradesh	102
190	Anantnag	J&K	97
191	Badgam	J&K	194
192	Bandipore	J&K	330
193	Baramula	J&K	182
194	Doda	J&K	15
195	Ganderbal	J&K	53
196	Jammu	J&K	120
197	Kargil	J&K	41

198	Kathua	J&K	57
199	Kishtwar	J&K	103
200	Kulgam	J&K	218
201	Kupwara	J&K	181
202	Leh(Ladakh)	J&K	50
203	Pulwama	J&K	145
204	Punch	J&K	121
205	Rajouri	J&K	43
206	Ramban	J&K	35
207	Reasi	J&K	0
208	Samba	J&K	341
209	Shupiyan	J&K	962
210	Srinagar	J&K	137
211	Udhampur	J&K	141
212	Bokaro	Jharkhand	30
213	Chatra	Jharkhand	56
214	Deoghar	Jharkhand	56
215	Dhanbad	Jharkhand	29
216	Dumka	Jharkhand	109
217	Garhwa	Jharkhand	51
218	Giridih	Jharkhand	39
219	Godda	Jharkhand	47
220	Gumla	Jharkhand	158
221	Hazaribagh	Jharkhand	73
222	Jamtara	Jharkhand	83
223	Khunti	Jharkhand	144
224	Kodarma	Jharkhand	50
225	Latehar	Jharkhand	115
226	Lohardaga	Jharkhand	118
227	Pakur	Jharkhand	126
228	Palamu	Jharkhand	70
229	Pashchimi Singhbhum	Jharkhand	111
230	Purbi Singhbhum	Jharkhand	59
231	Ramgarh	Jharkhand	65
232	Ranchi	Jharkhand	78
233	Sahibganj	Jharkhand	77
234	Saraikela-Kharsawan	Jharkhand	77
235	Simdega	Jharkhand	108
236	Bagalkot	Karnataka	48
237	Bangalore	Karnataka	59
238	Bangalore Rural	Karnataka	78
239	Belgaum	Karnataka	104
240	Bellary	Karnataka	155
241	Bidar	Karnataka	53
242	Bijapur	Karnataka	55
243	Chamarajanagar	Karnataka	44
244	Chikkaballapura	Karnataka	63
245	Chikmagalur	Karnataka	58
246	Chitradurga	Karnataka	89
247	Dakshina Kannada	Karnataka	89

248	Davanagere	Karnataka	108
249	Dharwad	Karnataka	209
250	Gadag	Karnataka	65
251	Gulbarga	Karnataka	165
252	Hassan	Karnataka	60
253	Haveri	Karnataka	79
254	Kodagu	Karnataka	64
255	Kolar	Karnataka	56
256	Koppal	Karnataka	70
257	Mandya	Karnataka	73
258	Mysore	Karnataka	85
259	Raichur	Karnataka	127
260	Ramanagara	Karnataka	81
261	Shimoga	Karnataka	95
262	Tumkur	Karnataka	86
263	Udupi	Karnataka	83
264	Uttara Kannada	Karnataka	46
265	Yadgir	Karnataka	74
266	Alappuzha	Kerala	13
267	Ernakulam	Kerala	34
268	Idukki	Kerala	45
269	Kannur	Kerala	19
270	Kasargod	Kerala	24
271	Kollam	Kerala	101
272	Kottayam	Kerala	118
273	Kozhikode	Kerala	65
274	Malappuram	Kerala	37
275	Palakkad	Kerala	20
276	Pathanamthitta	Kerala	13
277	Thiruvananthapuram	Kerala	28
278	Thrissur	Kerala	43
279	Wayanad	Kerala	61
280	Lakshdweep	Lakshdweep	208
281	Alirajpur	Madhya Pradesh	191
282	Anuppur	Madhya Pradesh	392
283	Ashoknagar	Madhya Pradesh	243
284	Balaghat	Madhya Pradesh	181
285	Barwani	Madhya Pradesh	187
286	Betul	Madhya Pradesh	169
287	Bhind	Madhya Pradesh	134
288	Bhopal	Madhya Pradesh	359
289	Burhanpur	Madhya Pradesh	252
290	Chhatarpur	Madhya Pradesh	92
291	Chhindwara	Madhya Pradesh	199
292	Damoh	Madhya Pradesh	161
293	Datia	Madhya Pradesh	161
294	Dewas	Madhya Pradesh	105
295	Dhar	Madhya Pradesh	131
296	Dindori	Madhya Pradesh	169
297	Guna	Madhya Pradesh	80

298	Gwalior	Madhya Pradesh	134
299	Harda	Madhya Pradesh	113
300	Hoshangabad	Madhya Pradesh	102
301	Indore	Madhya Pradesh	106
302	Jabalpur	Madhya Pradesh	435
303	Jhabua	Madhya Pradesh	102
304	Katni	Madhya Pradesh	282
305	Khandwa (East Nimar)	Madhya Pradesh	111
306	Khargone (West Nimar)	Madhya Pradesh	148
307	Mandla	Madhya Pradesh	208
308	Mandsaur	Madhya Pradesh	97
309	Morena	Madhya Pradesh	123
310	Narsimhapur	Madhya Pradesh	437
311	Neemuch	Madhya Pradesh	157
312	Panna	Madhya Pradesh	206
313	Raisen	Madhya Pradesh	171
314	Rajgarh	Madhya Pradesh	81
315	Ratlam	Madhya Pradesh	89
316	Rewa	Madhya Pradesh	181
317	Sagar	Madhya Pradesh	235
318	Satna	Madhya Pradesh	155
319	Sehore	Madhya Pradesh	79
320	Seoni	Madhya Pradesh	204
321	Shahdol	Madhya Pradesh	302
322	Shajapur	Madhya Pradesh	97
323	Sheopur	Madhya Pradesh	170
324	Shivpuri	Madhya Pradesh	108
325	Sidhi	Madhya Pradesh	219
326	Singrauli	Madhya Pradesh	149
327	Tikamgarh	Madhya Pradesh	97
328	Ujjain	Madhya Pradesh	153
329	Umaria	Madhya Pradesh	171
330	Vidisha	Madhya Pradesh	236
331	Ahmadnagar	Maharashtra	29
332	Akola	Maharashtra	66
333	Amravati	Maharashtra	52
334	Aurangabad	Maharashtra	51
335	Bhandara	Maharashtra	30
336	Bid	Maharashtra	14
337	Buldana	Maharashtra	18
338	Chandrapur	Maharashtra	45
339	Dhule	Maharashtra	40
340	Gadchiroli	Maharashtra	43
341	Gondiya	Maharashtra	37
342	Hingoli	Maharashtra	13
343	Jalgaon	Maharashtra	22
344	Jalna	Maharashtra	13
345	Kolhapur	Maharashtra	38
346	Latur	Maharashtra	19
347	Mumbai	Maharashtra	97

348	Mumbai Suburban	Maharashtra	0
349	Nagpur	Maharashtra	163
350	Nanded	Maharashtra	23
351	Nandurbar	Maharashtra	76
352	Nashik	Maharashtra	52
353	Osmanabad	Maharashtra	22
354	Parbhani	Maharashtra	8
355	Pune	Maharashtra	49
356	Raigarh	Maharashtra	36
357	Ratnagiri	Maharashtra	22
358	Sangli	Maharashtra	50
359	Satara	Maharashtra	31
360	Sindhudurg	Maharashtra	62
361	Solapur	Maharashtra	29
362	Thane	Maharashtra	26
363	Wardha	Maharashtra	97
364	Washim	Maharashtra	7
365	Yavatmal	Maharashtra	35
366	Bishnupur	Manipur	255
367	Chandel	Manipur	150
368	Churachandpur	Manipur	147
369	Imphal East	Manipur	80
370	Imphal West	Manipur	317
371	Senapati	Manipur	207
372	Tamenglong	Manipur	529
373	Thoubal	Manipur	97
374	Ukhrul	Manipur	332
375	East Garo Hills	Meghalaya	311
376	East Khasi Hills	Meghalaya	200
377	Jaintia Hills	Meghalaya	212
378	Ribhoi	Meghalaya	227
379	South Garo Hills	Meghalaya	590
380	West Garo Hills	Meghalaya	317
381	West Khasi Hills	Meghalaya	253
382	Aizawl	Mizoram	97
383	Champhai	Mizoram	335
384	Kolasib	Mizoram	40
385	Lawngtlai	Mizoram	195
386	Lunglei	Mizoram	113
387	Mamit	Mizoram	40
388	Saiha	Mizoram	220
389	Serchhip	Mizoram	67
390	Dimapur	Nagaland	217
391	Kiphire	Nagaland	192
392	Kohima	Nagaland	133
393	Longleng	Nagaland	99
394	Mokokchung	Nagaland	132
395	Mon	Nagaland	58
396	Peren	Nagaland	54
397	Phek	Nagaland	194

398	Tuensang	Nagaland	88
399	Wokha	Nagaland	156
400	Zunheboto	Nagaland	152
401	Anugul	Odisha	197
402	Balangir	Odisha	122
403	Baleshwar	Odisha	108
404	Bargarh	Odisha	77
405	Baudh	Odisha	81
406	Bhadrak	Odisha	48
407	Cuttack	Odisha	122
408	Debagarh	Odisha	158
409	Dhenkanal	Odisha	78
410	Gajapati	Odisha	168
411	Ganjam	Odisha	186
412	Jagatsinghapur	Odisha	83
413	Jajapur	Odisha	98
414	Jharsuguda	Odisha	65
415	Kalahandi	Odisha	203
416	Kandhamal	Odisha	287
417	Kendrapara	Odisha	97
418	Kendujhar	Odisha	69
419	Khordha	Odisha	45
420	Koraput	Odisha	260
421	Malkangiri	Odisha	286
422	Mayurbhanj	Odisha	139
423	Nabarangapur	Odisha	223
424	Nayagarh	Odisha	122
425	Nuapada	Odisha	301
426	Puri	Odisha	88
427	Rayagada	Odisha	180
428	Sambalpur	Odisha	327
429	Subarnapur	Odisha	255
430	Sundargarh	Odisha	111
431	Karaikal	Puducherry	32
432	Mahe	Puducherry	49
433	Pudducherry	Puducherry	45
434	Yanam	Puducherry	0
435	Amritsar	Punjab	310
436	Barnala	Punjab	89
437	Bathinda	Punjab	104
438	Faridkot	Punjab	429
439	Fatehgarh Sahib	Punjab	159
440	Firozpur	Punjab	195
441	Gurdaspur	Punjab	70
442	Hoshiarpur	Punjab	44
443	Jalandhar	Punjab	63
444	Kapurthala	Punjab	71
445	Ludhiana	Punjab	85
446	Mansa	Punjab	80
447	Moga	Punjab	44

448	Muktsar	Punjab	133
449	Patiala	Punjab	212
450	Rupnagar	Punjab	109
451	Sahibzada Ajit Singh Nagar	Punjab	155
452	Sangrur	Punjab	64
453	Shahid Bhagat Singh Nagar	Punjab	105
454	Tarn Taran	Punjab	35
455	Ajmer	Rajasthan	179
456	Alwar	Rajasthan	158
457	Banswara	Rajasthan	243
458	Baran	Rajasthan	174
459	Barmer	Rajasthan	123
460	Bharatpur	Rajasthan	160
461	Bhilwara	Rajasthan	96
462	Bikaner	Rajasthan	128
463	Bundi	Rajasthan	133
464	Chittaurgarh	Rajasthan	123
465	Churu	Rajasthan	198
466	Dausa	Rajasthan	110
467	Dhaulpur	Rajasthan	208
468	Dungarpur	Rajasthan	220
469	Ganganagar	Rajasthan	104
470	Hanumangarh	Rajasthan	110
471	Jaipur	Rajasthan	102
472	Jaisalmer	Rajasthan	67
473	Jalor	Rajasthan	131
474	Jhalawar	Rajasthan	209
475	Jhunjhunun	Rajasthan	166
476	Jodhpur	Rajasthan	170
477	Karauli	Rajasthan	158
478	Kota	Rajasthan	275
479	Nagaur	Rajasthan	120
480	Pali	Rajasthan	208
481	Pratapgarh	Rajasthan	244
482	Rajsamand	Rajasthan	327
483	Sawai Madhopur	Rajasthan	136
484	Sikar	Rajasthan	117
485	Sirohi	Rajasthan	273
486	Tonk	Rajasthan	140
487	Udaipur	Rajasthan	298
488	East District	Sikkim	202
489	North District	Sikkim	376
490	South District	Sikkim	309
491	West District	Sikkim	152
492	Ariyalur	Tamil Nadu	28
493	Chennai	Tamil Nadu	76
494	Coimbatore	Tamil Nadu	117
495	Cuddalore	Tamil Nadu	39
496	Dharmapuri	Tamil Nadu	44
497	Dindigul	Tamil Nadu	21

498	Erode	Tamil Nadu	43
499	Kancheepuram	Tamil Nadu	40
500	Kanniyakumari	Tamil Nadu	28
501	Karur	Tamil Nadu	9
502	Krishnagiri	Tamil Nadu	25
503	Madurai	Tamil Nadu	123
504	Nagapattinam	Tamil Nadu	25
505	Namakkal	Tamil Nadu	22
506	Perambalur	Tamil Nadu	59
507	Pudukkottai	Tamil Nadu	43
508	Ramanathapuram	Tamil Nadu	31
509	Salem	Tamil Nadu	91
510	Sivaganga	Tamil Nadu	19
511	Thanjavur	Tamil Nadu	86
512	The Nilgiris	Tamil Nadu	50
513	Theni	Tamil Nadu	49
514	Thiruvallur	Tamil Nadu	39
515	Thiruvarur	Tamil Nadu	80
516	Thoothukkudi	Tamil Nadu	53
517	Tiruchirappalli	Tamil Nadu	92
518	Tirunelveli	Tamil Nadu	72
519	Tiruppur	Tamil Nadu	28
520	Tiruvannamalai	Tamil Nadu	55
521	Vellore	Tamil Nadu	37
522	Viluppuram	Tamil Nadu	82
523	Virudhunagar	Tamil Nadu	38
524	Adilabad	Telangana	60
525	Hyderabad	Telangana	124
526	Karimnagar	Telangana	36
527	Khammam	Telangana	95
528	Mahbubnagar	Telangana	52
529	Medak	Telangana	46
530	Nalgonda	Telangana	43
531	Nizamabad	Telangana	37
532	Rangareddy	Telangana	9
533	Warangal	Telangana	49
534	Dhalai	Tripura	104
535	North Tripura	Tripura	217
536	South Tripura	Tripura	100
537	West Tripura	Tripura	93
538	Agra	Uttar Pradesh	182
539	Aligarh	Uttar Pradesh	128
540	Allahabad	Uttar Pradesh	276
541	Ambedkar Nagar	Uttar Pradesh	344
542	Auraiya	Uttar Pradesh	397
543	Azamgarh	Uttar Pradesh	122
544	Baghpat	Uttar Pradesh	140
545	Bahraich	Uttar Pradesh	140
546	Ballia	Uttar Pradesh	26
547	Balrampur	Uttar Pradesh	153

548	Banda	Uttar Pradesh	179
549	Bara Banki	Uttar Pradesh	202
550	Bareilly	Uttar Pradesh	62
551	Basti	Uttar Pradesh	226
552	Bijnor	Uttar Pradesh	161
553	Budaun	Uttar Pradesh	264
554	Bulandshahr	Uttar Pradesh	149
555	Chandauli	Uttar Pradesh	158
556	Chitrakoot	Uttar Pradesh	147
557	Deoria	Uttar Pradesh	344
558	Etah	Uttar Pradesh	487
559	Etawah	Uttar Pradesh	329
560	Faizabad	Uttar Pradesh	395
561	Farrukhabad	Uttar Pradesh	173
562	Fatehpur	Uttar Pradesh	106
563	Firozabad	Uttar Pradesh	84
564	Gautam Buddha Nagar	Uttar Pradesh	73
565	Ghaziabad	Uttar Pradesh	67
566	Ghazipur	Uttar Pradesh	240
567	Gonda	Uttar Pradesh	41
568	Gorakhpur	Uttar Pradesh	272
569	Hamirpur	Uttar Pradesh	119
570	Hardoi	Uttar Pradesh	154
571	Jalaun	Uttar Pradesh	265
572	Jaunpur	Uttar Pradesh	170
573	Jhansi	Uttar Pradesh	156
574	Jyotiba Phule Nagar	Uttar Pradesh	375
575	Kannauj	Uttar Pradesh	185
576	Kanpur Dehat	Uttar Pradesh	84
577	Kanpur Nagar	Uttar Pradesh	101
578	Kanshiram Nagar	Uttar Pradesh	141
579	Kaushambi	Uttar Pradesh	254
580	Kheri	Uttar Pradesh	126
581	Kushinagar	Uttar Pradesh	83
582	Lalitpur	Uttar Pradesh	261
583	Lucknow	Uttar Pradesh	971
584	Mahamaya Nagar	Uttar Pradesh	18
585	Mahoba	Uttar Pradesh	147
586	Mahrajganj	Uttar Pradesh	219
587	Mainpuri	Uttar Pradesh	44
588	Mathura	Uttar Pradesh	191
589	Mau	Uttar Pradesh	329
590	Meerut	Uttar Pradesh	154
591	Mirzapur	Uttar Pradesh	90
592	Moradabad	Uttar Pradesh	152
593	Muzaffarnagar	Uttar Pradesh	126
594	Pilibhit	Uttar Pradesh	304
595	Pratapgarh	Uttar Pradesh	114
596	Rae Bareli	Uttar Pradesh	107
597	Rampur	Uttar Pradesh	248

598	Saharanpur	Uttar Pradesh	160
599	Sant Kabir Nagar	Uttar Pradesh	236
600	Sant Ravidas Nagar (Bhadohi)	Uttar Pradesh	224
601	Shahjahanpur	Uttar Pradesh	118
602	Shrawasti	Uttar Pradesh	302
603	Siddharthnagar	Uttar Pradesh	194
604	Sitapur	Uttar Pradesh	278
605	Sonbhadra	Uttar Pradesh	312
606	Sultanpur	Uttar Pradesh	443
607	Unnao	Uttar Pradesh	334
608	Varanasi	Uttar Pradesh	218
609	Almora	Uttarakhand	57
610	Bageshwar	Uttarakhand	0
611	Chamoli	Uttarakhand	113
612	Champawat	Uttarakhand	202
613	Dehradun	Uttarakhand	180
614	Garhwal	Uttarakhand	45
615	Hardwar	Uttarakhand	131
616	Nainital	Uttarakhand	82
617	Pithoragarh	Uttarakhand	94
618	Rudraprayag	Uttarakhand	65
619	Tehri Garhwal	Uttarakhand	110
620	Udham Singh Nagar	Uttarakhand	88
621	Uttarkashi	Uttarakhand	61
622	Bankura	West Bengal	112
623	Barddhaman	West Bengal	104
624	Birbhum	West Bengal	103
625	Dakshin Dinajpur	West Bengal	104
626	Darjiling	West Bengal	253
627	Haora	West Bengal	121
628	Hugli	West Bengal	61
629	Jalpaiguri	West Bengal	125
630	Koch Bihar	West Bengal	114
631	Kolkata	West Bengal	146
632	Maldah	West Bengal	97
633	Murshidabad	West Bengal	137
634	Nadia	West Bengal	104
635	North Twenty Four Parganas	West Bengal	43
636	Paschim Medinipur	West Bengal	70
637	Purba Medinipur	West Bengal	38
638	Puruliya	West Bengal	82
639	South Twenty Four Parganas	West Bengal	68
640	Uttar Dinajpur	West Bengal	77
