# Supplementary material for: Estimates and correlates of district-level maternal mortality ratio in India
Source: PLOS Glob Public Health. 2022 Jul 18;2(7):e0000441. doi: 10.1371/journal.pgph.0000441 (PMC10021851; doi:10.1371/journal.pgph.0000441)
Supplement: S4 Table — (PDF) [file pgph.0000441.s005.pdf]

S 4 Table. Comparison of Infant Mortality Rate, Sex Ratio at Birth and Crude Birth Rate from SRS and HMIS

Indicator	SRS (2018)	HMIS (2017-19)
Infant Mortality Rate	32	26.2
Sex Ratio at Birth	111	108
Crude Birth Rate	20.2	24
