# Supplementary material for: Estimates and correlates of district-level maternal mortality ratio in India
Source: PLOS Glob Public Health. 2022 Jul 18;2(7):e0000441. doi: 10.1371/journal.pgph.0000441 (PMC10021851; doi:10.1371/journal.pgph.0000441)
Supplement: Attachment — Submitted filename: Point-by-Point Response 05 April.docx [file pgph.0000441.s006.docx]

**Point-by-Point Response to Editor and Reviewers Comments**

To

Dr. Stephen J. MaCall, DPhil

Academic Editor, PLOS Global Public Health

Date: 03/26/2022

Dear Dr. Stephen,

Thank you for allowing us to revise and resubmit our paper "**Estimates and Correlates of District-Level Maternal Mortality Ratio in India**" [PGPH-D-22-00024]. From the comments section, we observe that all the three reviewers have given positive feedback but also expressed minor concerns and posted some queries as well. We have addressed the comments of the editor and reviewers as described below in order of their comments. **While we have uploaded the track-change version of the paper as well, some of the corrections were also made after creating a clean copy of the paper. We request the reviewers to give more attention to clean copy**. The responses are highlighted in blue colour.

**Editorial comments:**

**Comment 1:** Please amend your Financial Disclosure statement. If you did not receive any funding for this study, please simply state: "The authors received no specific funding for this work."

**Authors' response:** The suggestion is well taken, and we have added the financial disclosure as suggested by the editor.

**Comment 2:** Please update your Competing Interests statement. If you have no competing interests to declare, please state: "The authors have declared that no competing interests exist."

**Authors' response:** The suggestion is well taken, and we have added the competing interest statement as suggested by the editor.

**Comment 3:** Please provide us with a direct link to the base layer of the map used in Figures 1, 2, and 3, and ensure this location is also included in the figure legend.

**Authors' response:** The suggestion is well taken, and we have provided the base layer of the map for Figures 1, 2 and 3. The direct link to the base layer of the map is **[https://github.com/datameet/maps/](https://github.com/datameet/maps/)**.

The link states and we quote: "Data License: Unless explicitly stated, all datasets in this repository is shared under **CC BY 4.0 license**. Please mention and link to relevant dataset in the attribution, e.g. India boundaries by DataMeet India community (**CC BY 4.0**)".

**Reviewer 1**

**Comment 4:** The table 2, requires appropriate titles for the columns. The column 'Obs' does not signify whether the value is for number of districts, or the the absolute number for each of the variable.

**Authors' response:** The suggestion is well taken, and we have amended the titles of the columns.

**Comment 5:** There are different factors operating for IMR & MMR. IMR as proxy measure for MMR does not suit.

**Authors' response:** Thank you for your comment. We agree that the factors are not exactly the same; however, several previous studies have established a systematic and close association between these measures, at least at the macro-level. Also, a few studies have derived MMR using IMR levels for specific geographies where the MMR data is not available. Please refer to the below studies.

**Bhat PM, Navaneetham K, Rajan SI. Maternal mortality in India: estimates from a regression model. *Studies in family planning*. 1995 Jul 1:217-32.**

Also, we have run a correlation between these two measures and presented them below. The level of ***r*** suggests a fairly good association.

**Comment 6:** Required to be mention in details, the method for district estimates were adjusted was done?

**Authors' response:** Thanks for this suggestion; we have extended the description of the method adopted.

**Comment 7:** Mention the cut off values used for categorization of high or low MMR.

**Authors' response:** These cut-off values are decided based on SDG targets for MMR. **See** **line no.-300 to 305** in our original and also in the revised draft.

**Comment 8:** The uniform nomenclature to be maintained either states and UTs or provinces.

**Authors' response:** The suggestion is well taken, and we have revised the paper accordingly.

**Comment 9:** Is there any significance of the information provided in the lines 309 to 311, if not; it can be removed.

**Authors' response:** These lines are regarding the cut-off values used for categorization of high or low MMR. We believe that they are essential. Your comment seven specifically asking for it.

**Comment 10:** The validity and reliability check was done for the MMR. Similarly it may be done for factors associated with MMR.

**Authors' response:** The data source used for other factors in the model are not from HMIS; they are compiled from other widely accepted and verified data sources such as the Census of India, Sample Registration System, and National Family Health Survey.

**Reviewer 2**

Many thanks for asking me review this paper assessing the sub-district level maternal mortality ratio in India using HMIS data. This is a very useful analysis for monitoring progress towards achieving the SDG MMR target. As India is a very large country with health devolved to state governments it is important to obtain disaggregated state/ district level data to assess the within country variability. The analysis is appropriate and the manuscript is by and large well written. I have a few suggestions to improve the manuscript:

**Comment 11:** The introduction is still very long and would suggest some of that can be moved to the discussion section. For example, the paragraph lines 111 to 122 will sit better in the discussion section.

**Authors' response:** The suggestion is well taken, and we have revised the paper accordingly. Information from lines **111 to 122** is now shifted to the discussion.

**Comment 12:** There is an assumption that reader is familiar with the sample registration system, HMIS etc. It will be useful to get a description of these data sources - particularly these two with a description of how the data are collected.

**Authors' response:** The suggestion is well taken, and we have extended the HMIS data description **[see line no 156-174].**

**Comment 13:** Some clarification regarding the limitations of SRS data and how HMIS can overcome these limitations would be good.

**Authors' response:** We have now added this information. SRS provides information only for major states of India on key demographic indicators. While HMIS provides information on a large number of health indicators for all states and all districts of India. . This has been written in the paper; **see lines 128 to 140.**

**Comment 14:** The methods section states that data triangulation was done using several different databases. However, table 1 does not include for example, census data. Please could you clarify how this data was used? On the same note, the link for data only appears to give HMIS data. Can the authors please confirm that it will be possible to repeat all the analyses reported in the paper using this data alone?

**Authors' response:** Yes, it is possible to repeat the analyses very easily. One reason why we kept our method simple is, to make the possibility that any public health scholar can repeat this exercise. We have uploaded all the excel files related to the study along with the revised paper. Moreover, all the data we used are openly accessible in the public domain.

**Comment 15:** Please state the strengths and limitations of the study in the discussion section. In particular please mention that HMIS will only collect facility based data and that the determinants analysis is essentially an ecological study and therefore prone to ecological bias.

**Authors' response:** The suggestion is well taken, and we have revised the paper accordingly. **See line no. -524 to 527.**

**Comment 16:** It will be good to report any monitoring of maternal deaths such as Confidential Enquiries into Maternal Deaths at national, state and health facility level. if not, future recommendation for automated HMIS data to feed into such enquiries can be suggested.

**Authors' response:** So far, a direct evaluation of maternal deaths reported in HMIS has been conducted through Population Research Centers (PRCs). One of our co-authors' is also involved in such an exercise [see Salve PS, Naiker SK, Golandaj JA, *et al.* **Situational analysis of maternal death review in India: Evidence from health management information system**. *Child Youth Serv Rev* 2020;**119**:105723. doi:https://doi.org/10.1016/j.childyouth.2020.105723].

However, we have assessed the quality of the HMIS data to the extent possible through secondary sources and also took inferences from previous evaluations such as Sharma et al. (2016) for the state of Haryana (**see lines 162-165**). In any post-enumeration surveys, it is not possible to assess a rare event like maternal death, which requires a large sample. An all-India-level sample survey like NFHS with more than 6,00,000 samples couldn't bring out a reliable data on maternal deaths; how can a post enumeration survey do it. Thus, we used other indicators assessed by Sharma et al. (2016) for the inferences on the quality of HMIS data.

The district-level relationship between MMR and other maternal health care, health infrastructure, and socio-demographic factors presented through linear regression analyses show the expected direction of the relationship, thereby validating our MMR estimates. The expected direction of the relationship reveals that our MMR estimates coincide with the district's maternal health care coverage, demographic and socioeconomic status. If there was a health facility-led bias in MMR registration, we would not have seen the expected direction of the relationship between these variables.

**Reviewer 3**

A very interesting study. I applaud the authors for their hard work and analysis. I recommend publication if my comments can be addressed in the subsequent revision.

**Comment 17:** Epidemiological studies are moving away from presenting models that adjust for all variables in the same model (although it is still very prevalent in the literature) unless these variables are theoretically underpinned as this can lead to erroneous results. For example, poor household economic status changed direction with MM after adjusting for other variables. Does this mean that poor household economic status is not correlated or that other variables explain the relationship between poor HH economic status and MM? To avoid this error the analysis needs to present (1) univariable analysis (2) multivariable models for each "MM vs. factor relationship" adjusting only for variables that can be confounders, ideally informed by causal diagrams.

References: Westreich, Daniel, and Sander Greenland. "The table 2 fallacy: presenting and interpreting confounder and modifier coefficients." American journal of epidemiology 177.4 (2013): 292-298.

Griffith, Gareth J., et al. "Collider bias undermines our understanding of COVID-19 disease risk and severity." Nature communications 11.1 (2020): 1-12. It is likely more appropriate to present each variables in a univariable model rather than presenting a fully adjusted model.

Laubach, Zachary M., et al. "EIC (Expert Information Criterion) not AIC: the cautious biologist's guide to model selection." arXiv preprint arXiv:2010.07506 (2020).

Tennant, Peter WG, et al. "Use of directed acyclic graphs (DAGs) to identify confounders in applied health research: review and recommendations." International journal of epidemiology 50.2 (2021): 620-632.

**Authors' response:** Thank you for the positive feedback. We agree with your argument about multi-collinearity issues in the regression analysis and subsequent erroneous results.

Except for model 7, we have ensured that strongly correlated variables such as "poor economic status of households and Years of schooling" are included in the same model.

The wrong direction of the results for poor economic status in model 7 is due to the inclusion of years of schooling. Thus, we have re-did model 7 by taking care of the collinearity problem.

We have also added the univariate results to our OLS regression Table. The previous draft also had Univariate regression analyses as a part of Geo-Spatial regressions.

On the other hand, we expect a strong correlation between ANCs, PNCs, and Institutional deliveries. But, in the Indian context, we don’t find this. Therefore, we have included those variables together in the same model.

However, drawing causal models in itself becomes a separate paper. Therefore, currently, we are not including it here. The references mentioned above are beneficial for planning our future work that exclusively focuses on identifying causal factors.

**Comment 18:** Can you specify in the manuscript the proportion of the population where IMR was used as a proxy? Can the justification of the use of IMR be further expanded in the methods section?

**Authors' response:** They are 17 states, but they are smaller states together and comprise less than 5% of the population of India. They all together comprise 116 out of 640 districts. We have added this information in the revised manuscript.

**Comment 19:** Please can the introduction be reduced and the limitations of the data sources be expanded.

**Authors' response:** The suggestion is well taken, and we have revised the paper accordingly.

**Comment 20:** Lots of abbreviations in the manuscript - please can you reduce these to make the paper more readable.

**Authors' response:** Some of them are unavoidable, but we have well taken the suggestion and revised the paper accordingly. In the revised manuscript, we kept essential abbreviations related to data and variable names that came multiple times in the paper.

**Comment 21:** Tables - make sure they can be read without referring to the paper (make sure abbreviations are all explained in the footnotes)

**Authors' response:** The suggestion is well taken, and we have revised the paper accordingly.

**We have addressed or responded to all the comments and suggestions given by all THREE reviewers. I hope the revised paper addressed the concerns of all reviewers. We have consulted several demographers and public health researchers to arrive at this regularly replicable methodology for monitoring district-level MMR estimates. We believe our response satisfies all THREE reviewers. We are more than happy to address any further concerns**

Yours Sincerely,

Authors.
